# No foreign language effect in Schizotypy: Evidence from German-English bilinguals^☆^

**DOI:** 10.1016/j.scog.2025.100410

**Published:** 2025-12-01

**Authors:** Steven Samuel, Markus Boeckle

**Affiliations:** a Department of Psychology, School of Health & Medical Sciences, City St. George's University of London, University of London, UK; b Karl Landsteiner University of Health Sciences, Dr. Karl-Dorrek-Straße 30, 3500, Krems, Austria; c D.O.T. Research Group for Mental health of Children and Adolescents, Ludwig Boltzmann Society at Karl Landsteiner University of Health Sciences, Krems, Austria; d Department of Psychiatry and Psychotherapeutic Medicine, University Hospital Tulln, Alter Ziegelweg 10, 3430, Tulln, Austria

**Keywords:** Schizotypy, foreign language effect, Bilingualism, Schizophrenia, German

## Abstract

Previous research has suggested that fewer schizophrenic and schizotypal traits are reported in a second language than a mother tongue. Such results make sense in the light of the so-called Foreign Language Effect (FLE), whereby bilinguals make more rational decisions and are less influenced by emotions and biases in a learned second language (L2) than a mother tongue (L1). However, this previous research is to date very limited, and apart from one large-scale quantitative study is based primarily on anecdotal evidence. In the study reported here, we gave German-English bilinguals the Schizotypal Personality Questionnaire in either English or German. If doing the questionnaire in an L2 (here English) makes participants think more rationally and less emotionally, then fewer schizotypal traits should be reported in these participants than those who answer the same questions in their L1 (German). Results failed to support this hypothesis; there was no evidence that bilinguals reported fewer traits in their second language. We interpret these data as suggesting that the link between schizotypy (specifically) and language context may be weaker or less reliable than hitherto supposed.

## 1 Introduction

Schizophrenia is a condition whose root cause is currently unknown (Dugan, 2014), and which affects approximately 0.4 %–0.7 % of the world population (Saha et al., 2005). It is associated with multiple positive and negative symptoms, including but not limited to auditory hallucinations, delusions of control, and the insertion, withdrawal, and broadcast of thoughts (Frith, 2005; Levy et al., 2010; McGuire et al., 1993; Waters et al., 2012). Individuals with schizophrenia often suffer deficits and/or abnormalities in metacognition and social cognition, and report lower social quality of life than healthy individuals (Frith, 2004; Hasson-Ohayon et al., 2015; Langdon et al., 2001). Even high schizotypy, a multidimensional construct that underlies schizophrenia and is also expressed in healthy individuals (Kwapil and Barrantes-Vidal, 2015; Wong and Raine, 2020) defined as a “latent personality organization that putatively indicates an individual's liability to psychosis/schizophrenia” ((Bora, 2020), p. 97),has been associated with social cognitive difficulties (Bora, 2020; Langdon and Coltheart, 2001).

Any means of alleviating the difficulties associated with schizophrenia or high schizotypy would likely prove beneficial to affected individuals' quality of life. Switching from a mother tongue into a second language context may provide such a possibility. Some intriguing findings emerged in the latter half of the Twentieth Century which suggested that bilingual patients with schizophrenia demonstrate remarkable differences when interviewed in a second language rather than their mother tongue. In a study of 30 patients, Hemphill (1971) reported that “a patient may be frankly psychotic and express delusions in one of his language but appear to be non-psychotic when he thinks and converses in another.” (p.1391), adding that a patient may even *deny* having hallucinations if they are asked in a second language. This effect has been reported in other research groups, each of which have reported that patients either appear non-psychotic to practitioners, believe themselves to be symptom-free when acting in a second language, or both (De Zulueta et al., 2001; Del Castillo, 1970; Paradis, 2008). It has even been claimed that second language training can result in early discharge of patients (Matulis, 1977).

These findings fit neatly within what is now known as the Foreign Language Effect (FLE). The FLE is the name given to the phenomenon by which (healthy) bilinguals make different decisions in a foreign language relative to a mother-tongue context (Gao et al., 2015; Keysar et al., 2012). For example, bilinguals are less likely to exaggerate their income in a foreign language than their native tongue (Bereby-Meyer et al., 2020), and tend to act with fewer intuitive biases and less emotionality in a second- than first-language context (Costa et al., 2014). In one study, Keysar et al. (2012) investigated the ‘framing effect’ in the context of bilingualism, contrasting logically equivalent dilemmas posed as either ‘gain-frame’ (save 200,000 people, or save either 0 or 600,000 according to chance), or ‘loss-frame’ (400,000 people will die, or you could save 0 or 600,000 according to chance). They found that bilinguals were less likely to gamble in the gain-frame than loss-frame scenarios, but only in their mother tongue. They interpreted this as the foreign language reducing ‘description dependency’, allowing more rational, less biased decisions to be made.Indeed Hemphill, 14, appeared to presage the FLE when claiming that patients were more rational in a second language than their mother tongue. This more psychologically distanced, less heuristically-biased form of thinking has been attributed to the fact that a second language is typically learned in a more formal environment, leading to less affective processing. For example, bilinguals experience greater reactions to emotion-laden and taboo words in a first than second language (Dewaele, 2004). The FLE also fits within a dual-process framework for decision making, with one system being fast, automatic, and based on affective content (i.e., the mother tongue); the other slow, reflective, and based on more neutral content (Kahneman and Frederick, 2002).

It is perhaps surprising that, decades since the conjecture of Hemphill (1971) little research has been done on bilinguals' self-reports of schizophrenic symptoms and schizotypal traits in their first and second languages, and none linking this to the FLE. Indeed, there are further reasons to believe that there could be a significant relationship between bilingualism and schizophrenia, based on reports of unusual interactions between bilinguals' languages and their experiences of symptoms. In a review of the literature, Paradis (2008) noted that bilingual patients may become impaired in their ability to use one language specifically when they are acutely psychotic (Oquendo, 1996), and they may experience auditory hallucinations in one language alone. Hemphill (1971) reported that hallucinations were heard only in the mother tongue, such that people are heard to speak languages they do not know (such as the Queen of England speaking Afrikaans), though some report hallucinations predominantly in the second language (Ocejo et al., 1991). Others suggest auditory hallucinations are context-dependent,based on the patient's thought process at the time of the hallucination (Wang et al., 1998). There are also reports of hallucinations first in one language and then another after medication (Laski and Taleporos, 1977), and even hallucinations exclusively in a *third* language (Wang et al., 1998). The significance of these findings is that the experience of schizophrenia appears to vary across different languages in the same individual, which fits with the notion that language context and potentially proactively switching languages might also modulate symptoms.

A weakness of the early research is that evidence was small-scale, often case-study based. More recently, support has come from a large-scale quantitative investigation involving 222 patients. Using measurements that were obtained using the Brief Psychiatric Rating Scale (Lukoff et al., 1986), which involves a mixture of self-report and coding of behaviour by the interviewer, Brown and Weisman de Mamani (2017) found that patients reported more positive symptoms — specifically hallucinations, thought disturbances and suspiciousness — in a mother tongue context than second language context. However, no differences were found in measures of negative symptoms such as affect and anergia. Similar findings were reported by the same researchers in a large non-clinical sample (414 college students), each of whom completed a self-report assessment of schizotypy (O-LIFE: (Mason et al., 1995)). The data showed that those who completed the task in their mother tongue reported greater unusual experiences and cognitive disorganisation on the questionnaire. Again, no differences were found in measures of negative symptoms. This evidence supports the possibility that there is an FLE in the reporting of symptoms and traits in both schizophrenia *and* schizotypy. However, since all participants were tested in English, all those who had English as a second language were bilinguals, while those who had English as their first language could have been monolinguals, and therefore, any hidden differences between these two populations could have led to these results (a possibility that the authors proposed themselves when discussing potential limitations in their study). The authors also highlighted that patients in their study were not randomly assigned to a first- or second-language context but chose for themselves, and that 78 % of the clinical population chose their mother tongue. It is possible therefore that those who opted for an interview in their second language did so based on a feeling that their L2 would create the best perception of themselves in the interview setting, (e.g., Oquendo, 1996), or perhaps their L2 was in fact now their more dominant language, meaning the usual logic of the FLE did not apply. Additionally, it is not clear that those for whom English was a *second* language would interpret the English items of the O-LIFE in the same way that a native speaker would. These potential weaknesses were addressed in the present study.

### 1.1 The present study

In sum, the evidence for a relationship between schizophrenia/schizotypy and language context is promising but presently very limited (Brown and Weisman de Mamani, 2017; Seeman, 2016). In the study we present here, we aimed to build upon this research. We assessed whether German-English bilinguals of German or Austrian nationality would endorse more schizotypal traits in their first language, German, than their second language, English. We chose German-English bilinguals as native German speakers have yet to be studied in this context. We chose schizotypy rather than schizophrenia as it allows the testing of healthy adults (providing a larger sample size) and because evidence of a FLE has come from non-clinical samples. Additionally, *all* participants were bilinguals, and we adapted the schizotypy questionnaires to be as equivalent as possible in meaning across both languages. This meant that evidence of an FLE would be more difficult to attribute to extraneous factors.

## 2 Methods

### 2.1 Participants

Participants were required to be aged 18–45, have normal or corrected-to-normal eyesight, and be a native speaker of German but have good English as a second language (minimum 3 out of 5 on a self-rating proficiency scale, with 0 = ‘no knowledge’ and 5 = ‘native or native-like proficiency’). Pre-screening required that participants held either German or Austrian nationality to take part in order to maximise the likelihood that participants were in fact native speakers of German, particularly as participation was online. No patients with schizophrenia were knowingly recruited to our sample – these were the only criteria set. Participants were recruited using Prolific Academic (www.prolific.co.uk) and the survey was supported by the Qualtrics online participation platform (www.qualtrics.com).

Given the reports of noticeable differences in both self-assessment and diagnoses in patients with schizophrenia depending on language context (Hemphill, 1971), we conducted an a priori power analysis based initially on an 80 % chance to detect a *large* effect size (*d* = 0.8, alpha 0.05, two-tailed *t*-test). This found that 52 participants were required (26 for each version of the Schizotypal Personality Questionnaire or SPQ). This number formed the basis of Phase 1 of recruitment. However, since schizotypy might be less sensitive to language context than clinical symptoms, and given the weaker effects related to schizotypy found by Brown and Weisman de Mamani (2017), we planned to run a second phase to recruit an *extra* 76 participants which would create sufficient power for an 80 % chance of detecting a *medium* effect size (*d* = 0.5). We considered effects smaller than this not to be of interest. We therefore preregistered two circumstances under which we would also run Phase 2 rather than stop upon completion of Phase 1. Under the first condition, there would be no statistically significant difference between groups but the effect size found from Phase 1 must be at least medium (*d* = 0.5), which would suggest that Phase 2 would provide enough power to detect the relevant effect. Under the second condition, there *would* be a statistically significant difference, but the effect size would nevertheless be *smaller* than large (*d* = 0.8), suggesting a potential false positive given the reduced sample size relative to the effect. Under any other circumstance, data collection would stop, and the results would be reported. These details and our planned methods and analyses can be found in our preregistration document here: https://archive.org/details/osf-registrations-zdg6m-v1. The data used in the analysis can be found here: https://osf.io/6tfkz/files/uhk7y. Ethical approval was provided by the Plymouth University Research Ethics Committee. A total of 52 participants took part in this study (*M*_*Age*_ = 30, range 18–44, 22 females, one trans woman, 29 males), with only Phase 1 being mandated by the results. One more participant's data were excluded and replaced owing to a failed attention check. All but one gave German as their only first language (one gave German and Polish, in that order), and self-rated their proficiency as 5 out of 5. The average proficiency rating for English was 4.1 (range 3–5). No further language use or demographic information was collected beyond these data and no matching of the two groups on any other variable was attempted.

### 2.2 Materials and procedure

#### 2.2.1 Abridged SPQ and abridged SPQ-G

All participants completed specially adapted and abridged versions of *either* the German-language version of the Schizotypal Personality Questionnaire, German version (SPQ-G), which was translated by Klein et al. (1997) from the original English version, or an adapted and abridged version of the original English SPQ devised by Raine (1991). Participant assignment to the German or English version was random, and we utilized a between-subjects design so as not to allow participants to strategically carry over responses from one version of the questionnaire to the other and to avoid activation of the non-target language through an entirely single-language context. The advertisement for the study was in German, but after group allocation all information was in the target language. We chose the SPQ because it was recently reported to be the most common measure of schizotypy in a metanalysis of studies concerning Theory of Mind (Bora, 2020). The *original* SPQ and SPQ-G consist of 74 yes/no questions, each divided into nine subscales (‘No close friends’; ‘Constricted affect’; ‘Suspiciousness’; ‘Ideas of reference’; ‘Odd beliefs and magical thinking’; ‘Excessive social anxiety’; ‘Odd or eccentric behaviour’; ‘Unusual perceptual experiences’; ‘Odd speech’). Each ‘yes’ response is coded as one point on the scale, with higher scores reflecting higher schizotypy. However, some items in the original English SPQ use idiomatic language which may not carry the same nuance of meaning after translation into a different language (e.g. “drop hints”, “ramble on too much”, and to “have it in for” someone). As we were going to be comparing the results of the SPQ and SPQ-G, it was imperative that the items in both questionnaires were independently assessed for equivalence of meaning. Before testing participants, we therefore asked four native German speakers (academics) with English as a second language to compare the SPQ items across each language and sort each item into two categories: 1) those that carried the ‘same meaning across languages’, and 2) those that were not the same, or which they were unsure about. Of the 74 items, 43 were judged by all four raters to have the same meaning across languages. The results of this validation can be found in the supplementary materials. Many items were reported as meaning quite different things in the SPQ-G relative to the SPQ, as the figure of 43 out of 74 items implies. Note that for arguably the most important subscale, ‘Unusual Perceptual Experiences’, only two of the original seven items were removed through this process. These 43 were the only items we gave participants in the experiment. Finally, since the study was to be conducted online, we also inserted two attention check items, between items 10–11 and 50–51, where we simply instructed participants to select the bottom (‘No’/‘Nein’) and top (‘Yes’/‘Ja’) responses respectively (with the language displayed being the language of the rest of the questionnaire). All participants whose data were included in the final analysis passed these two checks. The study was presented using the Qualtrics online survey platform.

## 3 Results

### 3.1 Planned tests

As pre-registered, mean scores that exceeded more than 2.5 times the standard deviation of the group mean were Winsorized (set at the next highest observed mean within the set threshold). This was to ensure that outlying data points did not exert undue influence on our between-subjects design. The means of three participants were changed in this way, one in the English group and two in the German group (these were: An English score of 36 became 27; a German 32 and 27 each became 26). Excluding these individuals did not change the pattern of results or the direction of the data.

The mean abridged SPQ score was 14.5 (SE = 1.6) in English, and 12.2 (SE = 1.4) in German, a difference that was not statistically significant, *t*(1, 51) = 1.089, *p* = .28, *d* = 0.30. There was no evidence of deviation from normality in either cell (Shapiro-Wilks tests >0.05). Since the effect size was smaller than medium, we did not proceed to Phase 2. Average SPQ scores in a way that transposes perfectly onto means in previous research are not possible, but if we take the original two undergraduate samples analysed by Raine (1991) the averages were 26.9 and 26.3 from the complete 74-item survey. Expressed as a percentage these both round to 36 %. The means in the present study are 14.5 (L2) and 12.2 (L1) out of 43 items, 34 % and 28 % respectively. In sum, scores from the current sample are comparable if very slightly lower than these original values.

A Bayes Factor analysis using the Cauchy prior found the data were approximately twice as likely under the null hypothesis that no difference exists between groups (BF_10_ = 0.45). This did not meet our threshold of a null being three times more likely to be meaningful. However, this probability rose to almost *seven* times as likely under the null if the test concerned the specific and one-tailed hypothesis that scores should be higher in the first language (BF_10_ = 0.15).

We also performed a planned exploration of the individual items of the questionnaires to examine whether there was evidence of a pattern of differences particularly to one subscale or another. For brevity and clarity, we report these by subscale. Given the reduction in the number of items in the questionnaires and deviation from the original SPQ and SPQ-G, these data should be interpreted with caution, and we performed no statistical analyses. Contrary to the hypothesis, mean scores were higher in the L2 English context than the L1 German context on *every* subscale (see Table 1).

**Table 1:** Mean scores on the abridged SPQ by language and subscale.

	L1 German	L2 English
Abridged SPQ Score (total)	12.2	14.5
Constricted affect	1.42	2.04
Odd & eccentric behaviour	1.46	2.04
No close friends	0.85	1.31
Unusual perceptual experiences	0.96	1.27
Ideas of reference	2.04	2.19
Odd speech	2.31	2.38
Odd beliefs & magical thinking	0.12	0.19
Excessive social anxiety	1.54	1.58
Suspiciousness	1.73	1.77

## 4 Discussion

We found no support for a relationship between reported schizotypal traits and language context, as measured by carefully matched adaptations of the SPQ and SPQ-G. These latter results are not consistent with previous research showing a small but reliable decrease in schizotypal traits when adults complete questionnaires in a second language (Brown and Weisman de Mamani, 2017). Indeed, our results patterned in the opposite direction, with stronger traits endorsed in the second language, though never to a statistically significant degree. Overall, our findings suggest that the relationship between schizotypy and bilingualism may be weaker or less reliable than previously thought.

One account for our results is that the effect of a second language on self-reported symptoms and traits is simply weaker and/or less reliable in schizotypy as measured in healthy adults than schizophrenia. A more nuanced account may also be possible, albeit entirely speculative at this point. It could be that there is a difference between positive and negative symptoms and traits whereby *negative* symptoms might be more sensitive to second language context in schizotypy, but *positive* symptoms more sensitive to second language effects in schizophrenia. Positive symptoms such as hallucinations and delusions are those which have been reported to interact most reliably with language context in patients, but for the general population experience of hallucinations and delusions are likely to be rarer than negative symptoms such as loss of affect and difficulties in social contexts. The SPQ and other measures of schizotypal personality might therefore be better at picking up variation in negative symptoms, which are precisely those symptoms which show less evidence of an FLE. This may explain why second language effect in schizotypy has previously been found to be small (Brown and Weisman de Mamani, 2017), and in the case of the present study, non-existent. However, there are also hints that bilinguals might report *greater* negative symptoms in their second language, i.e. the opposite of what is typically found in people with a diagnosis. Unexpectedly, our data from the abridged SPQ patterned very slightly but consistently (across every subscale) in favour of greater schizotypal traits in the second rather than first language, though to reiterate there was no statistically significant effect of this pattern. Although this is relatively unusual in the (small) literature to date, it is not unheard of. Marcos et al. (1973) reported that Spanish-English bilingual patients reported greater negative symptoms in their acquired second language than their mother tongue. The large-scale study of schizotypy in the healthy adult population by Brown and Weisman de Mamani (2017) found no significant differences in reporting of negative symptoms in different language contexts, but the data also appear to suggest a subtle (but again non-significant) trend towards greater negative symptoms in the second language. Although non-significant patterns of data should be treated cautiously, this is particularly notable because it moves in the opposite direction of the (significant) effect of positive symptoms found in the same sample. In their study with patients, it is also notable that the effect sizes of the statistically significant contrasts were quite small (partial eta squared of .021–.032), and again on negative symptoms no differences were found. Future research could therefore explore the potential for a ‘flip’ in the second language effect, where a second language context is related to reduced positive symptoms in patients, but greater negative symptoms in the non-clinical population.

Finally, there are methodological considerations to take into account. A limitation of our study relative to that of Brown and Weisman de Mamani (2017) is that our sample was significantly smaller. Our study and theirs also diverge in terms of the schizotypy questionnaire given to participants; we used (adapted versions of) the SPQ and they used the O-LIFE. However, we also made changes that we felt would extend and improve upon their design, based in large part on the researchers' own assessment of their study's limitations. These included recruiting only bilinguals, and carefully selecting items that carried the same meaning across both languages. Overall, our design addressed these points because we recruited only German-English bilinguals who had good English proficiency, and only gave them items from the SPQ and SPQ-G which were externally validated as meaning the same thing across languages. Having made these changes, we found no evidence of differential reporting of traits between first and second languages.

In our power analysis, our choice of initial (large) effect size was based on the accounts of powerful differences described in the Introduction, by Hemphill (1971) and others. As this was based on a clinical population, whereas we included only healthy participants, we also allowed for a medium effect size if Phase 2 of data collection had been warranted. However, the only previous study conducted with a non-clinical sample, that by Brown and Weisman de Mamani (2017), reported only a small effect size (partial eta squared of .02). Our sample size was not sufficiently powered to detect a small sample size, and therefore our results should be treated with caution. However, given that the data not only failed to support the hypothesis but actually patterned (non-significantly) in the opposite direction to what was expected, it appears unlikely (though not impossible) that increasing the sample size would lead to a different outcome.

Is it possible that the reason we found no evidence of a second language effect is because our German-English bilingual samples did not feel that English was a ‘foreign’ language? Theoretically, it has been argued that decisions made using the slower, more deliberative system ‘migrate’ to the fast, intuitive system with practice (Kahneman and Frederick, 2002), and if we view English being or becoming over time an alternative ‘native’ language then we may have no a priori reason to assume any differences should arise. Overall, we feel it is highly unlikely that our samples viewed English as an alternative mother tongue. Firstly, in Germany and Austria English is neither an official language of state nor typical in the family home, but rather a language learned through schooling and media exposure. These are precisely the characteristics of a second language mentioned in the FLE and dual-process literature. Secondly, the mean self-reported English proficiency score was below the ‘native-like proficiency’ standard for a 5 out of 5 on the self-reported proficiency scale, meaning that our groups also did not feel that their English was as good as their German. In sum, we neither have reason to believe, nor data to show, that participants felt English was an alternative mother tongue.

## 5 Conclusion

Previous studies have found support for a second language effect related to schizophrenia and schizotypy, with fewer symptoms and traits reported in a second language context. Using a carefully controlled cross-linguistic measure of schizotypal traits, we found no convincing evidence a second language effect in schizotypy.

## Open data practices

The (anonymized) pre-registration documents for this experiment can be found here: https://osf.io/zdg6m/?view_only=6be66ce3a0174203a512bac294aee4c4.

## CRediT authorship contribution statement

**Steven Samuel:** Writing – review & editing, Writing – original draft, Validation, Project administration, Methodology, Investigation, Formal analysis, Data curation, Conceptualization. **Markus Boeckle:** Writing – review & editing, Validation, Methodology, Investigation, Conceptualization.

## Ethics statement

Ethical approval was provided by the Plymouth University Research Ethics Committee. All participants gave informed consent to participate in this study.

## Declaration of competing interest

None to report.

## Appendix A Supplementary data

Supplementary material 1 Image 1

Supplementary material 2 Image 2
